# miR-29a regulates the proliferation and differentiation of retinal progenitors by targeting Rbm8a

**DOI:** 10.18632/oncotarget.16669

**Published:** 2017-03-29

**Authors:** Yi Zhang, Bingqiao Shen, Dandan Zhang, Yuyao Wang, Zhimin Tang, Ni Ni, Xiaoliang Jin, Min Luo, Hao Sun, Ping Gu

**Affiliations:** ^1^ Department of Ophthalmology, Shanghai Ninth People's Hospital, Shanghai Jiaotong University School of Medicine, Shanghai 200011, China

**Keywords:** retinal progenitor cells, microRNA (miR)-29a, RBM8A, proliferation, differentiation

## Abstract

During development, tight regulation of the expansion of retinal progenitor cells (RPCs) and their differentiation into neuronal and glial cells is important for retinal formation and function. Our study demonstrated that microRNA (miR)-29a modulated the proliferation and differentiation of RPCs by suppressing RBM8A (one of the factors in the exon junction complex). Particularly, overexpression of miR-29a reduced RPC proliferation but accelerated RPC differentiation. By contrast, reduction of endogenous miR-29a elicited the opposite effects. Overexpression of miR-29a repressed the translation of Rbm8a, thus negatively regulating RPC proliferation and promoting the neuronal and glial differentiation of RPCs, and knockdown of endogenous Rbm8a phenocopied the observed effects of miR-29a overexpression. Furthermore, a luciferase reporter assay showed that miR-29a directly interacted with the Rbm8a mRNA 3′UTR, which indicated that Rbm8a is the direct target of miR-29a. To further verify the result, co-overexpression of the Rbm8a 3′ UTR-wt (plasmids into which the Rbm8a 3′ UTR sequence had been introduced) and miR-29a in RPCs rescued the phenotype associated with miR-29a overexpression, reversing the promotion of differentiation and inhibition of proliferation. These results show a novel mechanism by which miR-29a regulates the proliferation and differentiation of RPCs through Rbm8a.

## INTRODUCTION

Retinal progenitor cells (RPCs) are a subset of undifferentiated cells with indefinite potential for self-renewal and differentiation into retinal neuronal and glial lineages [1, 2]. Despite the presence of RPCs in adult mammalian eyes, a reliable and abundant source of RPCs is not currently available [3–5], and this limitation encourages further studies that explore the molecular mechanisms of how to improve RPC proliferative potential. Retinal neurons cannot self-repair after injury or exposure to pathological conditions [6, 7], emphasizing the importance of better understanding the mechanisms of controlling RPC differentiation. RPC self-renewal and differentiation is tightly regulated by a series of events including dynamic changes in the pattern of expression of microRNAs (miRNAs) that drive cellular fate determination [8, 9].

miRNAs, a class of small noncoding RNA molecules, which regulate gene expression by binding to partially complementary target sites in the 3′untranslated region (3′UTR) of mRNAs, trigger either mRNA degradation or translational repression [10, 11]. In general, one gene can be regulated by multiple miRNAs, and one miRNA may regulate multiple target genes [10, 11]. Recent studies have suggested that miR-29 family including three members: miR-29a, miR-29b, and miR-29c, has complex functions in various cells [12–15] and contributes to normal tissue differentiation [16] such as skeletal myogenesis [13], osteoblast differentiation [17], and neuron differentiation [18, 19]. miR-29a has been shown to be expressed in mesenchymal stem cells and to serve as an important regulator in the neuronal differentiation of mesenchymal stem cells by targeting REST [18]. For retina cells, a study detected that miR-29a expression changes through Müller glia dedifferentiation and CD73^+^ rod photoreceptor differentiation [19]. Other studies reported that miR-29a may regulated the MMP-2 protein level in RPE cells [20] and associated with high myopia [21]. However, whether miR-29a regulates the proliferation and differentiation of RPCs remains unknown.

RBM8A, one of the factors in the exon junction complex, is a ribonucleoprotein with an RNA binding motif that preferentially binds to mRNAs during splicing [26]. A previous study demonstrated that it was highly expressed in the subventricular zone of the early embryonic cortex and was an crucial regulator involved in keeping the undifferentiated and self-renewable cell state of cortical neural progenitor cells [27]. The role of RBM8A in RPC fate determination was investigated in the current study.

In this study, our data demonstrate the role of miR-29a in the regulation of RPC proliferation and differentiation by using miR-29a mimics and inhibitors of miR-29a. We identified Rbm8a as one of the direct targets of miR-29a in RPCs by luciferase analysis and further investigated the function of RBM8A in the regulation of RPC fate determination by performing overexpression and knockdown analyses. Furthermore, the concomitant overexpression of both Rbm8a 3′ UTR-wt and miR-29a in RPCs rescues the cellular phenotype associated with the overexpression of miR-29a in RPCs, indicating that Rbm8a lies downstream of miR-29a and regulates RPC proliferation and fate determination. Taken together, our data demonstrate that miR-29a acts as an important regulator in RPC proliferation and differentiation by directly targeting Rbm8a. This study provides us with a better understanding of the molecular mechanisms that govern the self-renewal and differentiation of RPCs.

## RESULTS

### The endogenous expression levels of miR-29a and RBM8A during RPC differentiation

**Figure 1:**
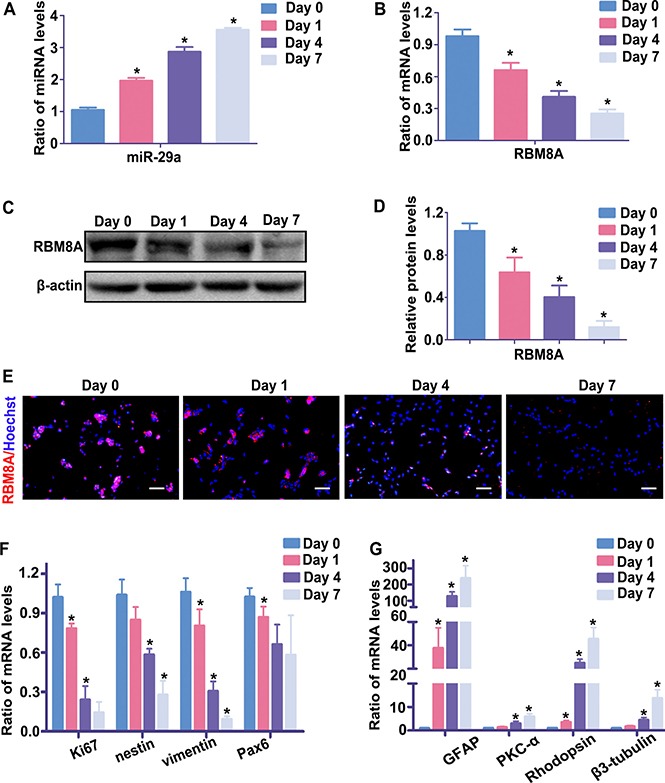
The endogenous expression levels of miR-29a and RBM8A during RPC differentiation (**A**, **B**) The qPCR results showed that the expression level of miR-29a gradually increased, whereas RBM8A expression exhibited the opposite trend during the seven-day differentiation of RPCs. (**C**, **D**, **E**) Western blotting and immunostaining results revealed that the protein level of RBM8A decreased over the same period. (**F**, **G**) qPCR showed that the expression of proliferative marker (Ki67), and retinal progenitor markers (nestin, Pax6 and vimentin) was gradually decreased, but the expression of RPC differentiation markers (Rhodopsin, a marker for photoreceptors; β3-tubulin, a pan-neuronal marker; PKC-α, a marker for bipolar neurons; and GFAP, a glial cell marker) had the reversed trend during RPC differentiation. Western bands were scanned and normalized to β-actin. Day 0 represents the undifferentiated RPC state and was used as a normalizer. Scale bars: 100 μm. Data are averages of three independent experiments. Error bars indicate the standard error of the mean. *P ≤ 0.05 (one-way ANOVA).

In previous reports, miR-29a was found to exert complex functions in various cells, including regulation of cell proliferation and differentiation. To reveal the function of miR-29a in RPCs, our study first investigated the expression of mi-29a during RPC differentiation. qRT-PCR results showed that miR-29a expression significantly increased after induction of RPC differentiation (RPCs cultured in differentiation medium), reaching an approximately 4-fold peak at day 7, compared with undifferentiated cells (RPCs cultured in proliferation medium) (day 0) (Figure 1A). Conversely, as shown in Figure 1B, the expression of Rbm8a, which was one of the potential miR-29a target genes predicted by TargetScan and microRNA software, progressively decreased during RPC differentiation. Western blotting and immunofluorescence was also performed to evaluate the protein levels of RBM8A, which decreased accordingly during RPC differentiation (RPCs cultured in differentiation medium) (Figure 1C, 1D and 1E). Meanwhile, qPCR detected that the expression of proliferative marker (Ki67), and retinal progenitor markers (nestin, Pax6 and vimentin) was gradual decreased, but the expression of RPC differentiation markers (Rhodopsin, a marker for photoreceptors; β3-tubulin, a pan-neuronal marker; PKC-α, a marker for bipolar neurons, and GFAP, a glial cell marker) had the reversed trend during RPC differentiation and thus it showed that RPCs were gradually differential over time (Figure 1F and 1G). These results suggested that there was a negative correlation of expression pattern of miR-29a and RBM8A during RPC self-renewal and differentiation.

### miR-29a inhibits RPC proliferation and accelerates RPC differentiation

**Figure 2:**
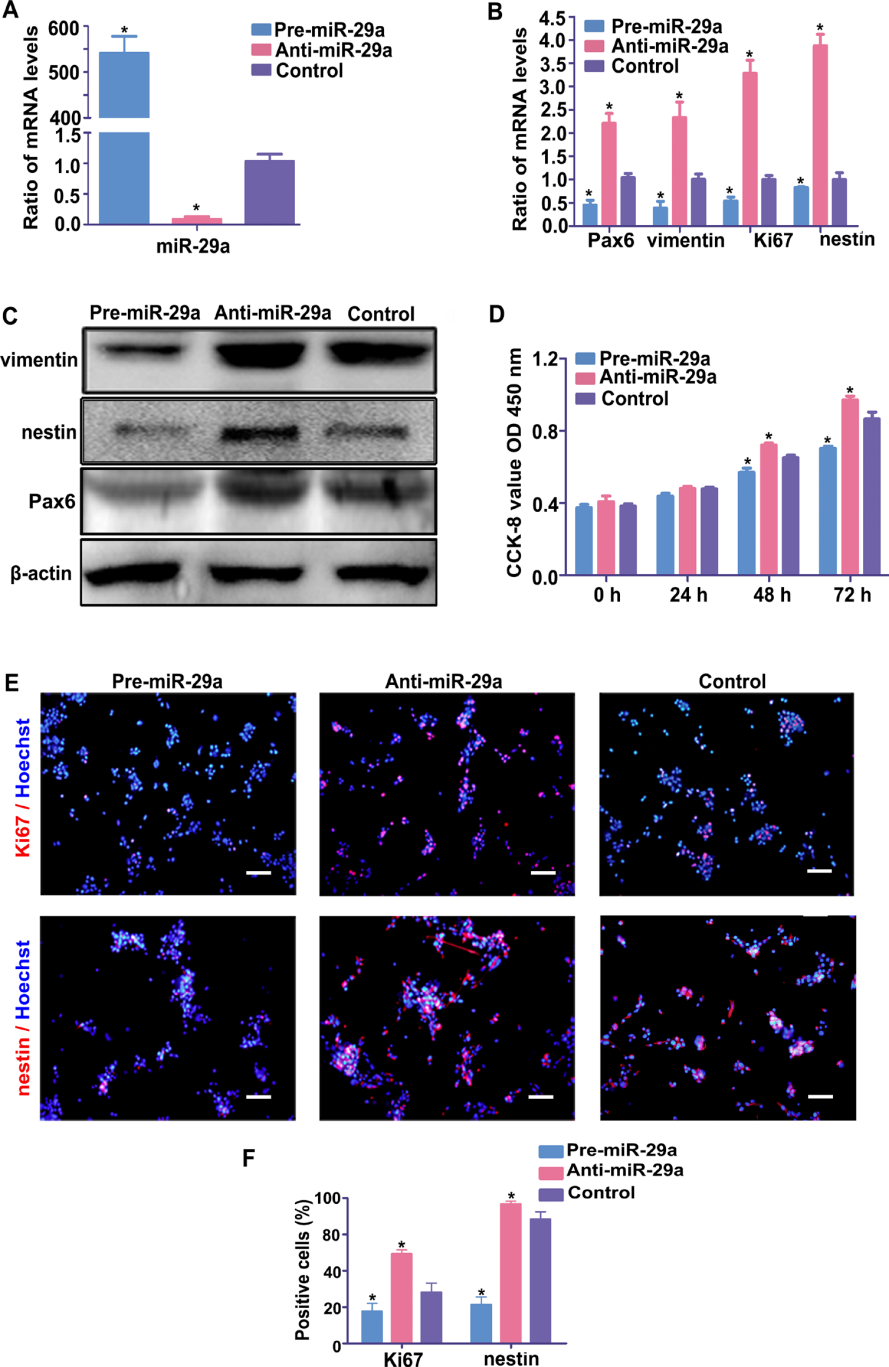
miR-29a inhibits RPC proliferation (**A**) The qPCR results revealed that the expression of miR-29a was sharply upregulated by transfection of RPCs with the miR-29a mimic and significantly downregulated by the miR-29a inhibitor treatment. (**B**) According to the qPCR analysis, the expression of Ki67 (a cell proliferation marker) and vimentin, Pax6 and nestin (retinal progenitor markers) decreased in the miR-29a mimic-treated RPCs and increased when the RPCs were treated with the miR-29a inhibitor. (**C**) The results of the western blotting analysis were consistent with the qPCR analysis. Western blotting bands were scanned and normalized to β-actin. (**D**) The proliferation ability of the RPCs was assessed via a CCK-8 analysis. The proliferation ability of the RPCs markedly increased when treated with the miR-29a inhibitor and decreased when treated with the miR-29a mimic under proliferation conditions. (**E**, **F**) Immunostaining with antibodies against Ki67 and nestin revealed the effects on RPC proliferation, and was consistent with the results shown above. These data are averages of three independent experiments. Scale bars: 100 μm. Data are averages of three independent experiments. Error bars indicate the standard error of the mean. *P ≤ 0.05 (Student's *t-test*).

To examine whether miR-29a regulates the proliferation and differentiation of RPCs, RPCs transfected with miR-29a mimics or inhibitors were cultured under proliferation or differentiation conditions. As shown in Figure 2A, the overexpression efficiency of the miR-29a mimic (pre-miR-29a group) or the inhibition efficiency of the miR-29a inhibitor (anti-miR-29a group) were detected by qPCR analysis. To evaluate the effect of miR-29a on RPC proliferation, RPCs treated with miR-29a mimic or inhibitor were cultured for 3 days under proliferation conditions before the cells were used for western blotting, qPCR, immunostaining and CCK8 analysis. The results of qPCR and western blotting showed a notable decrease in the nestin, Pax6 vimentin and Ki67 expression levels, which were attributable to the overexpression of miR-29a in the RPC cultures (Figure 2B and 2C). Conversely, miR-29a knockdown induced the opposite effect (Figure 2B and 2C). To verify the effects of miR-29a on RPC proliferation, a CCK8 analysis was also performed on RPC cultures, and these cells were treated with a miR-29a mimic or inhibitor under proliferation conditions. The data indicated no marked difference in the proliferation capacity between cultures treated with miR-29a mimics or inhibitor on the first day of culture; thereafter, inhibited expansion was recorded for RPC cultures treated with the miR-29a mimic, while the miR-29a inhibitor promoted the proliferation of RPCs (Figure 2D). After treating with miR-29a inhibitor (anti-miR-29a group), the percentage of Ki67-positive cells was higher than that in the control group (Figure 2E and 2F). By contrast, upregulation of miR-29a decreased the Ki67-positive cell percentage (pre-miR-29a group). Additionally, the percentage of nestin-immunoreactive cells decreased in RPCs treated with the miR-29a mimic and increased in those treated with the miR-29a inhibitor, compared with the control cells (Figure 2E and 2F). These results suggest that miR-29a can negatively regulate RPC proliferation.

**Figure 3:**
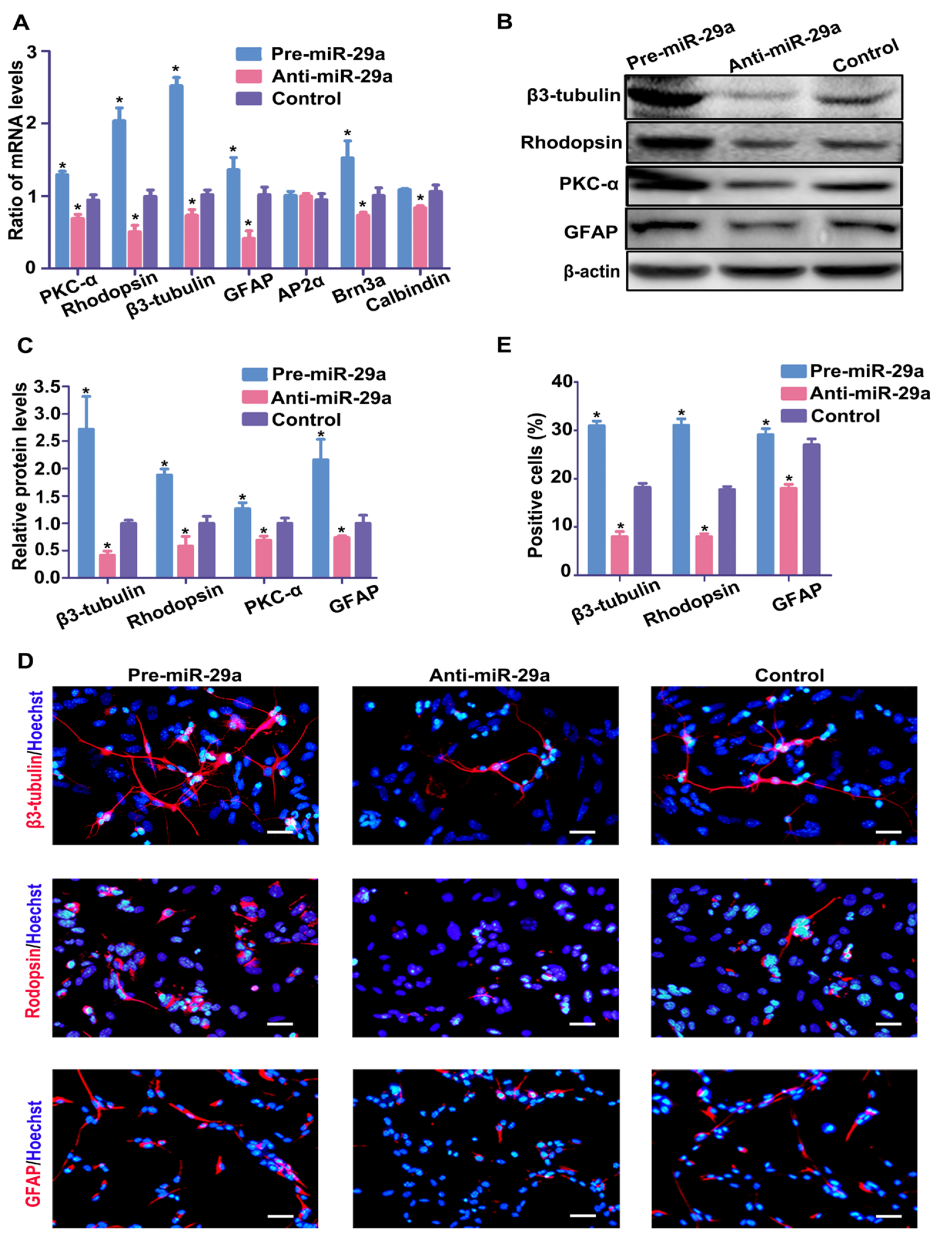
miR-29a promotes RPC differentiation (**A**–**C**) The expression levels of RPC differentiation-related markers, including PKC-α, Rhodopsin, β3-tubulin, Brn3a, calbindin and GFAP, were elevated by the miR-29a mimic but repressed by the miR-29a inhibitor according to qPCR and western blotting analyses. However, the expression of AP2α had no obvious change. Western blotting bands were scanned and normalized to β-actin. (**D**, **E**) The percentages of Rhodopsin-, β3-tubulin-, and GFAP-positive cells were evaluated to investigate RPC differentiation. The proportion of Rhodopsin- and β3-tubulin-positive cells was significantly augmented, and that of GFAP-positive cells slightly increased in the miR-29a mimic-treated RPC cultures. By contrast, the ratios of Rhodopsin, β3-tubulin and GFAP markedly decreased when treated with the miR-29a inhibitor. Retinal neuronal cell markers: PKC-α, Rhodopsin, β3-tubulin; glial cell marker: GFAP. Scale bars: 100 μm (for GFAP), 50 μm (for Rhodopsin and β3-tubulin). Data are averages of three independent experiments. Error bars indicate the standard error of the mean. *P ≤ 0.05 (Student's *t-test*).

Next, to determine the effect of miR-29a on the differentiation of RPCs, RPCs were transfected with the miR-29a mimic or miR-29a inhibitor and were subsequently induced to differentiate for 7 days (RPCs cultured in differentiation medium). The effects of miR-29a on RPC differentiation-related marker expression were evaluated by qPCR analysis. miR-29a mimic-treated RPC cultures exhibited higher levels of retinal neuronal cell markers (Rhodopsin, β3-tubulin, PKC-α, Brn-3a (a ganglion cell marker) and Calbindin (horizontal and ganglion cell marker)), and GFAP than the control cells (Figure 3A). However, when the cells were treated with the miR-29a inhibitor, the levels of these markers were downregulated (Figure 3A). The expression level of Ap2α (an amacrine cell marker) had no obvious changes (Figure 3A). Furthermore, in agreement with the qPCR results, western blotting results suggested that miR-29a expression had a marked effect on the protein markers of differentiation (Figure 3B and 3C). Immunostaining analyses indicated that, compared with control cells, in miR-29a mimic-treated RPC cultures, β3-tubulin and Rhodopsin were significantly increased and GFAP was slightly enhanced (Figure 3D and 3E). By contrast, the miR-29a inhibitor had the opposite effect (Figure 3D and 3E). These results indicate that miR-29a markedly enhances the differentiation of RPCs toward neuronal cells.

### *Rbm8a* is a target gene of miR-29a in RPCs

**Figure 4:**
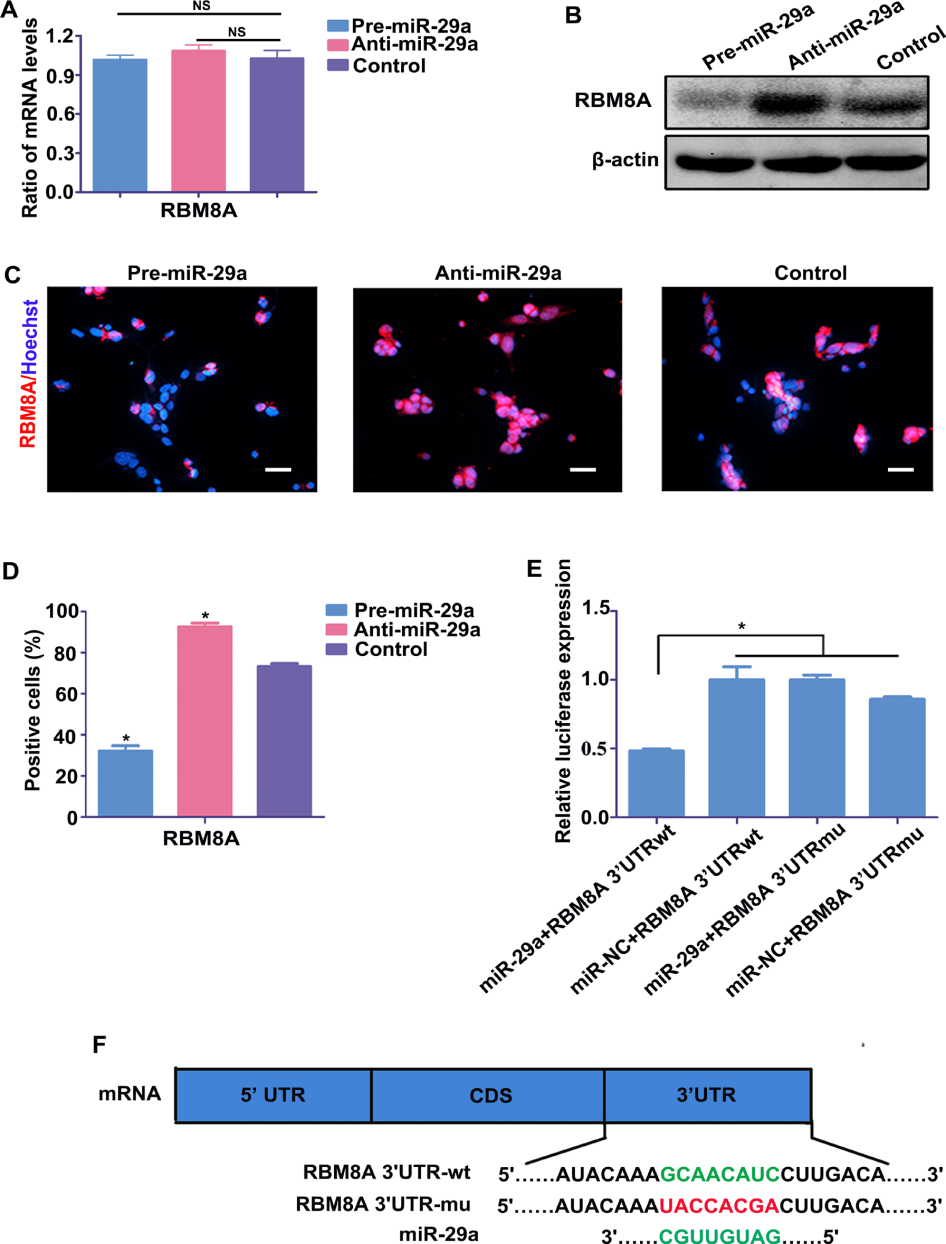
Rbm8a is a target gene of miR-29a in RPCs (**A**) qPCR showed that neither exogenous miR-29a nor the miR-29a inhibitor had effects on Rbm8a mRNA levels. (**B**–**D**) Western blotting and immunostaining analysis indicated that the RBM8A protein expression level was inhibited by the overexpression of miR-29a, but the downregulation of miR-29a via transfection with the miR-29a inhibitor promoted RBM8A protein expression. Western blotting bands were scanned and normalized to β-actin. Scale bars: 50 mm. (**E**) The dual luciferase reporter system indicated that the co-transfection of miR-29a and its wild type 3′-UTR binding site, termed Rbm8a 3′-UTRwt, dramatically reduced luciferase activity, whereas miR-29a had no effect on the mutated 3′-UTR binding region (Rbm8a 3′-UTRmu). The firefly luciferase activity data were all normalized to Renilla luciferase activity as a control. (**F**) Position 475-482 of the 3′-UTR of Rbm8a mRNA or a mutated sequence was designed and inserted into pGL3-control plasmids. Data are averages of three independent experiments. Error bars indicate the standard error of the mean. *P ≤ 0.05 (Student's *t-test* and one-way ANOVA ).

As shown in Figure 1, the negative correlation of expression pattern of miR-29a and RBM8A in RPCs undergoing differentiation and the prediction of Targetscan and microRNA software indicated that miR-29a might negatively regulate RBM8A to affect RPC cell determination. To test our hypothesis that Rbm8a is a direct target of miR-29a, RPCs were transduced with a miR-29a mimic or inhibitor in proliferation medium. The qPCR data showed no marked change in the mRNA expression level of Rbm8a in the miR-29a mimic or inhibitor treatment groups (Figure 4A). The results of western blotting showed that the protein level of RBM8A was repressed by miR-29a but promoted by the miR-29a inhibitor compared to the control (Figure 4B), suggesting that miR-29a regulates the expression of RBM8A by inhibiting translation. Furthermore, the immunostaining results exhibited an increased proportion of RBM8A-positive cells upon miR-29a inhibitor treatment, compared with control in RPC cultures under proliferation conditions (Figure 4C and 4D).

To further validate whether miR-29a directly interacted with the Rbm8a mRNA 3′UTR and subsequently interfered with the translation process, a luciferase reporter system was constructed. A 220 bp fragment of the Rbm8a 3′UTR containing either the wild-type or mutant sequences of position 475–482 was cloned downstream of the firefly luciferase coding sequence in the pGL3-control vector; these constructs were termed Rbm8a 3′UTR-wt and Rbm8a 3′UTR-mu, respectively. miR-29a mimic or control miRNA was individually co-transfected with Rbm8a 3′UTR-wt or Rbm8a 3′UTR-mu. Luciferase assays showed that the co-transfection of miR-29a and Rbm8a 3′UTR-wt dramatically decreased the luciferase activity compared with the other groups (Figure 4E), indicating that position 475–482 of the 3′UTR of Rbm8a was a direct target of miR-29a (Figure 4F).

Collectively, these data indicate that miR-29a directly binds to the Rbm8a 3′UTR and regulates RBM8A protein expression.

### RBM8A regulates RPC proliferation and differentiation

**Figure 5:**
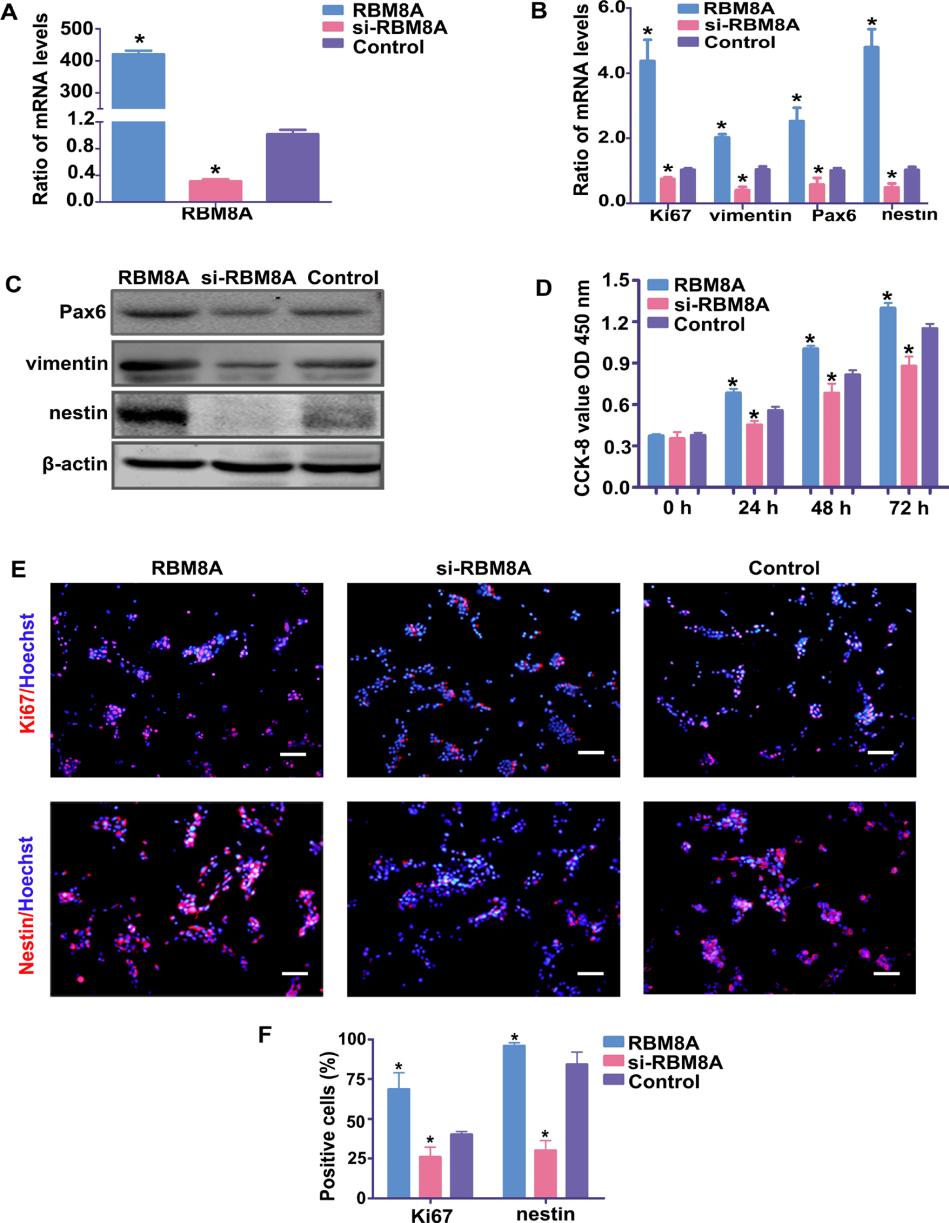
RBM8A enhances RPC proliferation (**A**) The qPCR results revealed that the expression level of Rbm8a decreased sharply with siRbm8a treatment but increased significantly in the Rbm8a clone-treated RPC cultures compared with the control. (**B**) The qPCR results showed that the expression levels of nestin, Pax6, vimentin and Ki67 decreased in siRbm8a -treated RPC cultures and increased in cells treated with the Rbm8a clone compared with control cells. (**C**) The protein expression levels of nestin, Pax6 and vimentin exhibited similar trends to the qPCR results. Western blotting bands were scanned and normalized to β-actin. (**D**) The proliferation ability of the RPCs transfected with siRbm8a or the Rbm8a clone was assessed via CCK-8 analysis. The expansion capacity of the cells markedly improved in the Rbm8a clone-treated cultures, whereas a weakened proliferation capacity was detected for the siRbm8a-treated cells. (**E**, **F**) The percentages of Ki67- and nestin-positive cells decreased in the siRbm8a-treated RPCs and increased when treated with the Rbm8a clone. Scale bars: 100 μm. Data are averages of three independent experiments. Error bars indicate the standard error of the mean. *P ≤ 0.05 (Student's *t-test*).

As a target gene of miR-29a, the ability of RBM8A to regulate proliferation and differentiation of RPCs was evaluated in the present study. The qPCR data indicated that the level of Rbm8a was markedly downregulated when RPCs were treated with siRbm8a but was upregulated to approximately 400-fold in Rbm8a-treated RPCs (Figure 5A). Under proliferation conditions (RPCs cultured in proliferation medium for 3 days), the qPCR results showed that the expression levels of nestin, Pax6, vimentin and Ki67 decreased in siRbm8a-treated RPC cultures and increased in cells treated with Rbm8a compared with control cells (Figure 5B). The protein expression levels of nestin, Pax6 and vimentin (Figure 5C) exhibited similar trends to the qPCR results. The CCK8 analysis also demonstrated a positive role for RBM8A in promoting RPC proliferation (Figure 5D). Furthermore, the immunostaining data revealed that siRbm8a-treated RPC cultures displayed fewer Ki67-positive and nestin-positive cells than the control cells (Figure 5E and 5F). Additionally, compared with the control cells, the overexpression of Rbm8a markedly increased the percentage of Ki67-positive cells and nestin-immunoreactive cells (Figure 5E and 5F).

**Figure 6:**
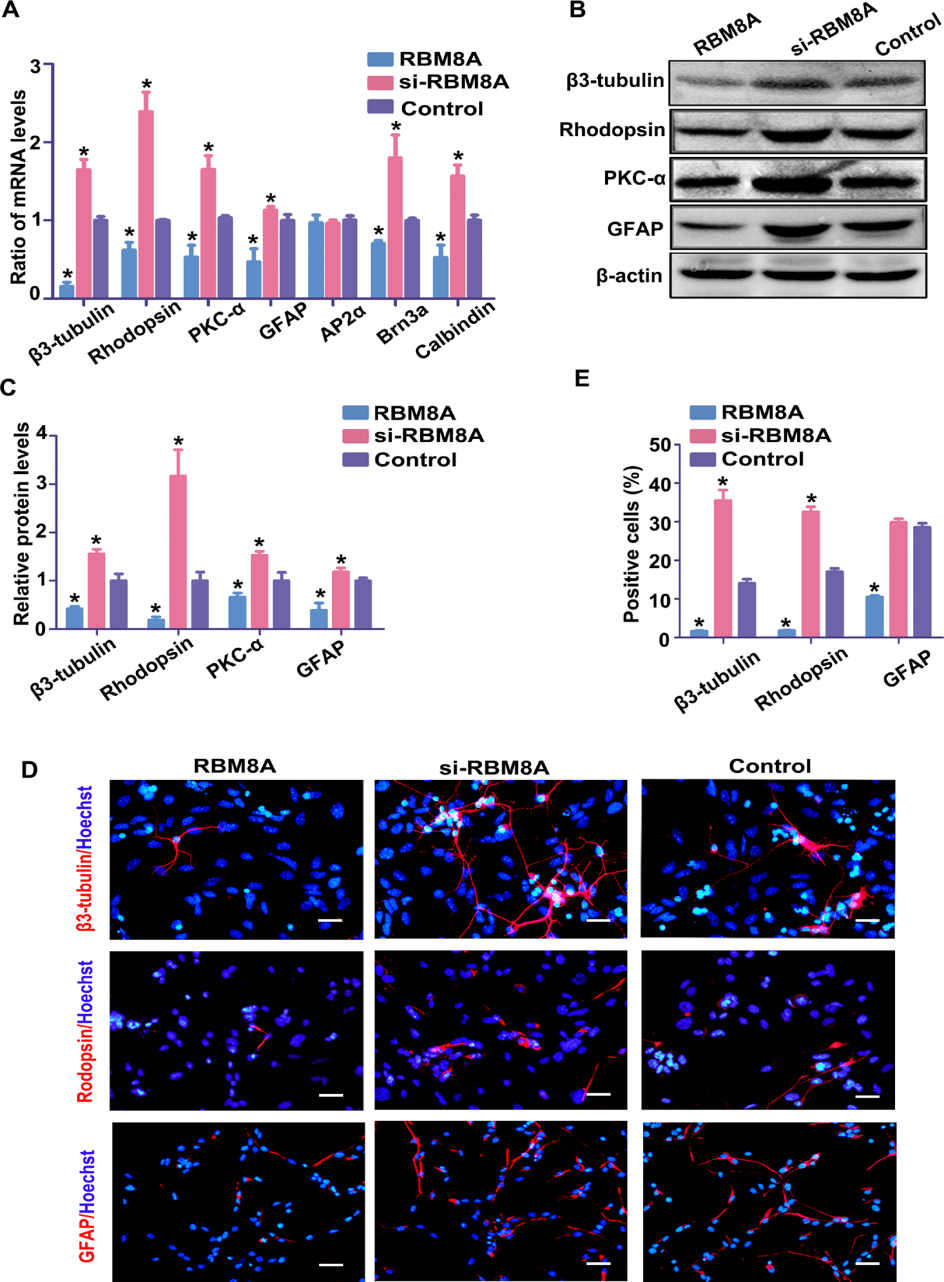
RBM8A reduces RPC differentiation (**A**–**C**) qPCR and western blotting analysis revealed large increases in the expression levels of Rhodopsin, β3-tubulin, PKC-α, GFAP, Brn3a, and calbindin in the RPC cultures, but their expression levels were significantly decreased in the Rbm8a-treated groups. The expression level of AP2α had no marked change. Western blotting bands were scanned and normalized to β-actin. (**D**, **E**) Immunostaining with antibodies against Rhodopsin, β3-tubulin and GFAP revealed that the proportions of Rhodopsin-, and β3-tubulin-immunoreactive cells were higher in the siRbm8a -treated cultures than in the control cells, but these positive percentages decreased upon Rbm8a clone treatment. Scale bars: 100 μm (for GFAP), 50 μm (for Rhodopsin and β3-tubulin). Data are averages of three independent experiments. Error bars indicate the standard error of the mean. *P ≤ 0.05 (Student's *t-test*).

To define the role of RBM8A in RPC differentiation, RPCs treated with the Rbm8a clones or siRbm8a were cultured under differentiation conditions for 7 days. Under siRBM8A treatment, the expression levels of Rhodopsin, β3-tubulin, PKC-α, GFAP, Brn3a and Calbindin in the RPC cultures demonstrated large increases by approximately 1.7-, 2.5-, 1.7-, 1.2-, 1.7- and 1.5- fold, respectively, according to the qPCR analysis, but their expression levels were decreased in the Rbm8a-treated groups (Figure 6A). A similar trend in the expression of neuronal and glial differentiation markers was observed by western blotting, when RPCs were treated with siRbm8a and the Rbm8a clone (Figure 6B and 6C). In addition, immunostaining analyses showed an increase in the number of these differentiation markers upon siRbm8a treatment, indicating the enhancement of neuronal differentiation, whereas a decrease upon Rbm8a clone treatment suggested the inhibition of differentiation (Figure 6D and 6E). These results suggest that RBM8A enhances RPC proliferation and inhibits their differentiation. These data suggest that RBM8A is an important regulator of RPC proliferation and differentiation.

### Rbm8a 3′UTR overexpression rescues the observed effects of miR-29a overexpression on RPC proliferation and differentiation

**Figure 7:**
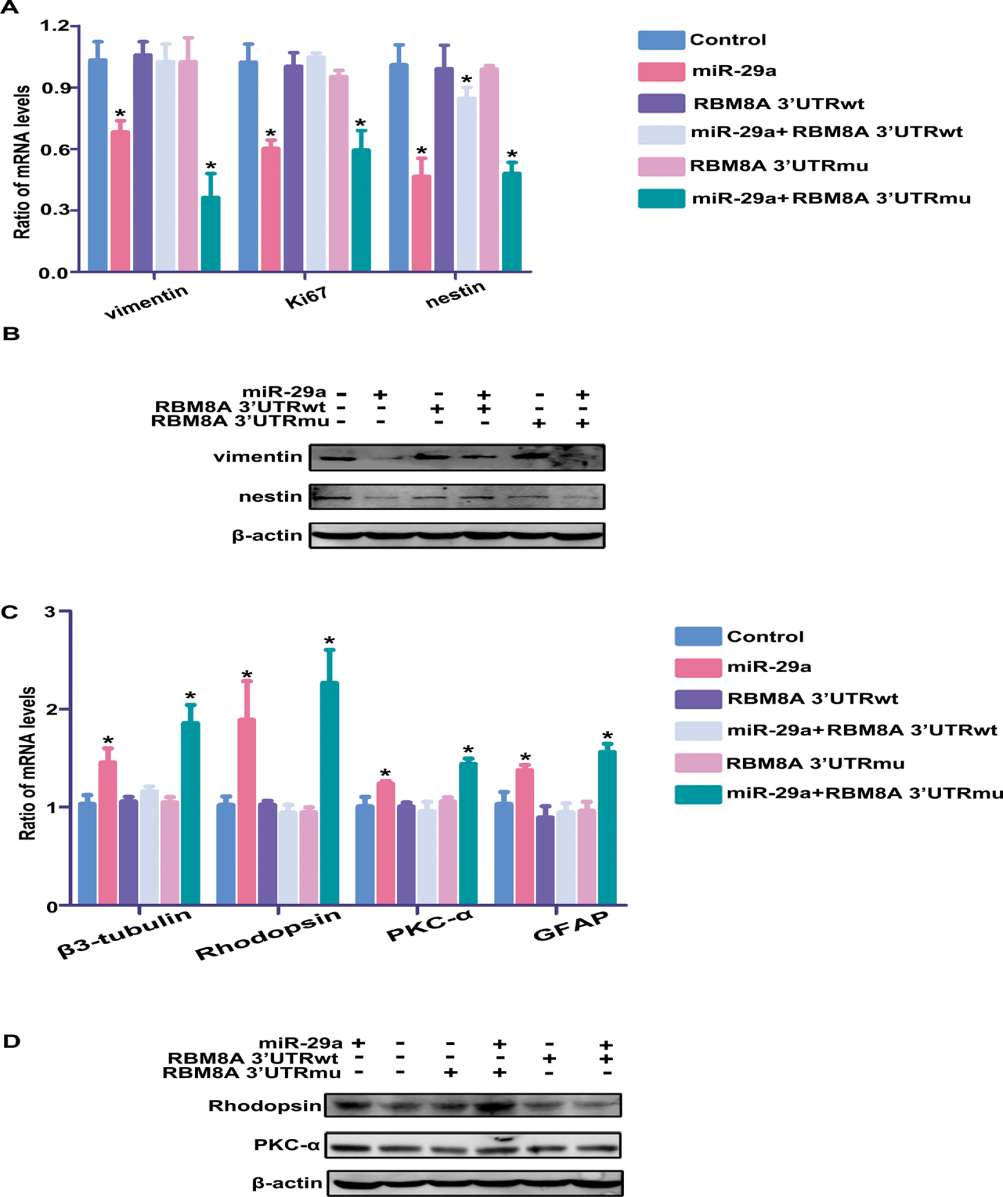
Rbm8a 3′-UTR overexpression rescues the observed effects of miR-29a overexpression on RPC proliferation and differentiation (**A**, **B**) qPCR and western blotting analysis indicated that Rbm8a 3′-UTRwt antagonizes the effects of miR-29a on RPC proliferation. However, the co-transfection of Rbm8a 3′-UTRmu with miR-29a induced no obvious change in RPC proliferation in comparison with transfection with only miR-29a. (**C**, **D**) Rbm8a 3′-UTRwt overexpression rescued the miR-29a-mediated increase in RPC differentiation-related markers, including Rhodopsin, β3-tubulin, PKC-α and GFAP. Data are averages of three independent experiments. Western blotting bands were scanned and normalized to β-actin. Error bars indicate the standard error of the mean. *P ≤ 0.05 (one-way ANOVA).

To determine whether the effect of miR-29a on RPC proliferation and differentiation was mediated through RBM8A, a miR-29a mimic was co-transfected with Rbm8a 3′UTR-wt or with Rbm8a 3′UTR-mut (these plasmids contained the Rbm8a 3′UTR sequence or a mutated Rbm8a 3′UTR sequence). qPCR results showed that Rbm8a 3′UTR-wt overexpression rescued the decrease in RPC proliferation (RPCs cultured in proliferation medium) that was caused by miR-29a overexpression (105% versus 60% for Ki67; 85% versus 47% for nestin; 103% versus 68% for vimentin, Figure 7A) and reversed the observed increase in neuronal and glial differentiation induced by miR-29a overexpression (RPCs cultured in differentiation medium) (116% versus 146% for β3-tubulin; 94% versus 189% for Rhodopsin; 96% versus 124% for PKC-α; 95% versus 137% for GFAP, Figure 7C). However, Rbm8a 3′UTR-mu could not reversed the effects of miR-29a on RPC proliferation and differentiation. In addition, under proliferation conditions, the results of the western blotting analysis (Figure 7B) showed that treatment with Rbm8a 3′UTR-wt rescued the proliferative inhibition induced by miR-29a overexpression. By contrast, the co-transfection of Rbm8a 3′UTR-mu with miR-29a showed no obvious change in RPC proliferation compared to transfection of miR-29a alone. Additionally, when RPCs were cultured under differentiation conditions, treatment with Rbm8a 3′UTR-wt successfully reversed the effects of miR-29a on RPC differentiation (Figure 7D).

**Figure 8:**
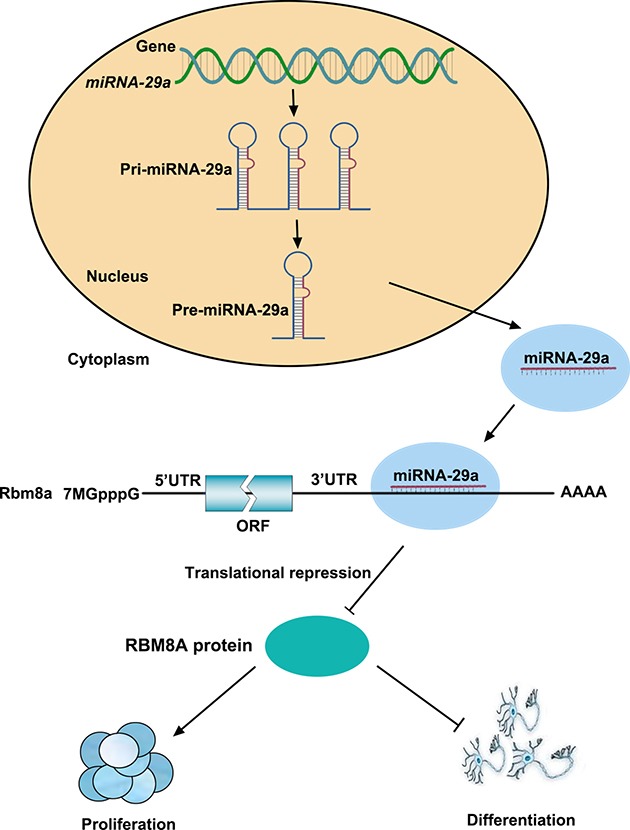
A model of the relationship between miR-29a and RBM8A in regulating RPCs proliferation and differentiation miR-29a inhibited RBM8A expression by binding to the Rbm8a 3′-UTR and, thus, negatively regulated RPC proliferation and positively regulated RPC differentiation.

Taken together, these results strongly suggest that miR-29a regulates the proliferation and differentiation of RPCs by targeting Rbm8a expression through a direct interaction with its 3′UTR (Figure 8).

## DISCUSSION

The results elucidate the molecular mechanism by which miR-29a regulates the proliferation and differentiation of RPCs by directly targeting Rbm8a. Methods to efficiently differentiate retinal cells from RPCs, which are available in abundance, would be likely to guarantee the development of successful cell-based therapy, which could be applied to various retinal dystrophies such as retinitis pigmentosa and age-related macular degeneration [28–30]. Hence, the identification of novel cellular and molecular players that modulate the self-renewal and differentiation of RPCs significantly impacts the development of cell therapy.

Recent data obtained by several groups supported a primary role of miRNAs in fine-tuning the signaling pathways that control stem cell development [31, 32]. The miR-29 family includes three mature members: miR-29a, miR-29b, and miR-29c [33, 34]. Mature miR-29s are highly conserved in humans, mice, and rats; share identical sequences at nucleotide positions 2–7 [34–36]; and are relevant in myogenic differentiation [12], skeletal myogenesis [13], osteogenesis [14, 15], and neuron differentiation [18]. The previous report detected changes of miR-29a expression in Müller glia-derived progenitors during the differentiation of a rod phenotype [19]. In the current study, we observed a new role of miR-29a in RPCs, and miR-29a expression was significantly upregulated during RPC differentiation. Our data demonstrated that miR-29a promotes the differentiation of RPCs, which induced the enhanced expression of RPC differentiation-related markers, including PKC-α, Rhodopsin and β3-tubulin. Additionally, a previous report showed that miR-29a plays a critical role in regulating the expression of p42.3, thus inhibiting cell proliferation in human gastric cancer [37]. Similarly, our results revealed that miR-29a serves as a negative regulator during the proliferation of RPCs. These data suggest that miR-29a plays an important role in governing the cell growth and fate determination of neural retina-derived progenitors.

A single miRNA usually has multiple target genes, and indeed, several target genes for miR-29a have been investigated, including REST [18], p42.3 [37], and YY1 [38]. In the present study, according to the predictions of microRNA (www.microrna.org) and TargetScan (www.targetscan.org) combined with our results, Rbm8a was revealed to serve as an important target of miR-29a in RPC proliferation and differentiation. RBM8A, one of the factors in the exon junction complex, controls mRNA stability and regulates mRNA splicing and translation [26]. A recent study reported that RBM8A was highly expressed in the subventricular zone of the early embryonic cortex and was crucial for the proliferation and differentiation of cortical neural progenitors [27]. Consistent with these findings, our data showed robust promotion of cell proliferation and inhibition of neuronal differentiation when Rbm8a was overexpressed in RPCs. By contrast, knockdown of Rbm8a had the opposite effect on RPCs. These data suggest that RBM8A acted as an important regulator by positively regulating RPC proliferation and negatively regulating RPC differentiation.

miRNA targets are preferentially expressed at high levels when expression of the targeting miRNA is low [18, 37, 39]. On the basis of our data, miR-29a expression gradually increased during RPC differentiation. By contrast, the expression level of its predicted target, RBM8A, was higher during the RPC proliferative stage and lower during differentiated stages. The inverse expression patterns of RBM8A and miR-29a indicated that miR-29a may function by turning off Rbm8a expression. It is known that miRNAs contribute to various developmental processes by acting as post-transcriptional regulators [31, 39, 40]. Our data demonstrated that knockdown of endogenous miR-29a resulted in increased expression of RBM8A at the protein level but not the mRNA level, which indicated that miR-29a regulated the expression of RBM8A by inhibiting its translation. In fact, miRNAs has been indicated to negatively modulate its target expression by directly targeting its 3′-UTR [41, 42]. According to our dual luciferase reporter assay, miR-29a bound to position 475-482 of the 3′-UTR of Rbm8a mRNA. As a result, miR-29a acted as an important upstream regulator to negatively modulate RBM8A expression by directly targeting its 3′ untranslated region (UTR). Furthermore, the forced expression of the 3′UTR-wt sequence of miRNA targets has been shown to compete with endogenous targets for miRNA binding, thus inhibiting the effects of miRNA on cell behavior [43]. We co-transfected miR-29a and Rbm8a 3′-UTR-wt into RPCs, which rescued the miR-29a-induced RPC proliferation deficiency and reversed the increased neuronal differentiation caused by miR-29a treatment alone. However, no marked changes in RPC proliferation and differentiation were observed upon co-transfection of miR-29a and Rbm8a 3′-UTR-mu compared with miR-29a treatment alone. These results indicate that the effect of miR-29a on RPC proliferation and differentiation was rescued by Rbm8a 3′-UTR. Taken together, our findings revealed that miR-29a inhibited RBM8A expression by binding to the Rbm8a 3′-UTR and, thus negatively regulated RPC proliferation and positively regulated RPC differentiation. Rbm8a is an excellent candidate as a miR-29a molecular target for regulating RPC proliferation and fate determination.

In summary, our data demonstrate that miR-29a regulates the proliferation and differentiation of RPCs by directly targeting Rbm8a, providing us with a better understanding of the molecular mechanisms that govern RPC proliferation and differentiation. Future studies to further elucidate the specific aspects of the roles of miR-29a and RBM8A in retinal development and function *in vivo* will be of great interest in this field.

## MATERIALS AND METHODS

### The isolation, culture and differentiation of RPCs

RPCs were harvested from fresh retinal tissue of postnatal-day-one GFP transgenic C57BL/6 mice (a gift from Dr. Masaru Okabe, University of Osaka, Japan). The cells were cultured with proliferation medium containing advanced Dulbecco′s Modified Eagle Media: Nutrient Mixture F-12 (DMEM/F12) (Invitrogen, Carlsbad, CA, USA), 1% N2 neural supplement (Invitrogen), 2 mM L-glutamine (Invitrogen), 100 U/ml penicillin-streptomycin (Invitrogen) and 20 ng/ml epidermal growth factor (EGF, Invitrogen) [44]. The culture medium was replaced every 2 days, and the cells were passaged at regular intervals of 3 or 4 days. For differentiation, the cells were cultured in differentiation medium containing 10% fetal bovine serum (FBS) (Invitrogen) without EGF. The cultures were then allowed to grow for seven days. The culture medium was renewed three times a week.

All animals were handled according to the ARVO animal usage standards and following approval by the animal care and use committee of the Schepens Eye Research Institute, where the original derivation of the cells was performed.

### miRNA, siRNA and plasmid construction

miR-29a oligonucleotides (miR-29a mimic, miR-29a inhibitor, and negative control) and small interfering RNAs (siRbm8a and negative control) were synthesized by Biomics Biotech Co.,Ltd. (Nantong, China). CMV-MCS-IRES-Cherry-SV40-Neomycinclone of Rbm8a was synthesized by Genechem Technology, Inc. (China). To generate the luciferase reporter vector, a 220-bp fragment of the Rbm8a mRNA 3′UTR containing the predicted miR-29a binding site (position 475–482) (Rbm8a mRNA 3′UTR-wt) and its mutant sequence (Rbm8a mRNA 3′UTR-mu) were synthesized by Genechem and cloned into pGL3-control vectors (Promega (Beijing) Biotech Co., Ltd., China).

### Transfection

For transient transfections, a 20 nM final concentration in Opti-MEM (Invitrogen) of miR-29a mimic, miR-29a inhibitor, negative control, siRbm8a, Rbm8a clone, Rbm8a 3′UTR-wt or Rbm8a 3′UTR-mu was blended with Lipofectamine 2000 (Invitrogen) in serum-free medium and incubated at room temperature for 20 min [45]. The mixture was then added to the cells in 24-well or 6-well plates at 80% fusion. The medium was renewed by proliferation or differentiation medium 6 h later. For long-term detection under differentiation culture conditions, RPCs were transfected every 3 days. The oligonucleotide sequence of siRbm8a was as follows: 5′-GUUGAAGGUUGGAUUCUCU-3′.

### Luciferase assay

HEK293 cells were transfected with either the miR-29a mimic or control miRNA in conjunction with the luciferase reporter constructs. Forty-eight hours after they were transfected, the cells were lysed and subjected to luciferase assays using a Dual Luciferase Reporter Assay System (Promega (Beijing) Biotech Co., Ltd., China) according to the manufacturer′s protocol.

### Immunocytochemistry

RPCs were plated on glass coverslips (VWR, West Chester, PA, USA) coated with laminin (Sigma-Aldrich, Saint Louis, MO, USA) in 24-well plates. Following transfection with miR-29a mimic, miR-29a inhibitor, siRbm8a or Rbm8a clone, cells were fixed with 4% paraformaldehyde (Sigma- Aldrich), and permeabilized with 1% Triton X-100 (Invitrogen) [46]. Then, The cells were incubated with an optimal concentration of rabbit polyclonal anti-RBM8A antibody (Novus; 1:100), mouse monoclonal anti-nestin (BD, San Jose, CA, USA; 1:200), mouse monoclonal anti-Ki-67 (BD; 1:200), mouse monoclonal anti-β3-tubulin (Chemicon, Billerica, MA, USA; 1:100), mouse monoclonal anti-Rhodopsin (Chemicon; 1:100) or mouse monoclonal anti-glial fibrillary acidic protein (GFAP) (Chemicon; 1:200) overnight at 4°C followed by an incubation with fluorescently labeled secondary antibodies (Alexa Fluor546-goat anti-mouse/rabbit, BD, San Jose, CA, USA; 1:800). After rinsing three times with PBS, nuclei were stained with 4′,6-diamidino-2-phenylindole (Hoechst; Invitrogen, Molecular Probes). Negative control samples were processed in parallel but without the primary antibody. Immunoreactive cells were visualized, and images were recorded using a fluorescence microscope (Olympus BX51, Japan).

### Western blotting analysis

After RPCs were transfected, total protein was obtained from the cells at specified time points. We performed Western blotting according to a standard protocol as previously described. The protein concentrations were detected using a BCA protein assay (Pierce, Rockford, IL, USA), and sodium dodecyl sulfate-polyacrylamide gel electrophoresis (SDS-PAGE) was used to separate the proteins, which were electroblotted onto PVDF membranes (Millipore, Billerica, MA, USA). The membranes were blocked with 5% nonfat milk and then incubated with rabbit polyclonal anti-RBM8A (Novus; 1:500), mouse monoclonal anti-nestin (BD; 1:1000), rabbit monoclonal anti-Pax6 (Biolegend; San Diego, CA, USA; 1:1000), rabbit monoclonal anti-vimentin (Epitomics; 1:1000), mouse monoclonal anti-β3-tubulin (Chemicon; 1:1000), mouse monoclonal anti-Rhodopsin (Chemicon; 1:200), mouse monoclonal anti-glial fibrillary acidic protein (GFAP) (Chemicon; 1:1000), mouse monoclonal anti-PKC-a (BD; 1:1000), and mouse anti-β-actin (Sigma, 1000) antibodies at 4°C for 8 h. Immunoreactive bands were detected using anti-rabbit (1:5000) or anti-mouse (1:5000) fluorescein-conjugated secondary antibodies (Abcam) and visualized by OdysseyV3.0 image scanning. Quantification of the densitometric intensities of the protein bands was performed using Bandscan 5.0 software, and the values of each sample were normalized to an endogenous reference (β-actin).

### RNA isolation, reverse transcription and quantitative polymerase chain reaction

The total RNA was harvested from the cultured cells with Trizol (Invitrogen) according to the manufacturer's instructions. The total amount of RNA was quantified by optical density (OD) measurements at OD260 nm and OD280 nm, and samples with OD260/280 nm ratios with a purity between 1.9 and 2.1 were used for cDNA synthesis. cDNA was obtained from the transcription of total RNA in a final reaction volume of 10 μL using a PrimeScript RT reagent kit (Perfect Real Time; TaKaRa) or a miRcute miRNA first-strand cDNA synthesis kit (Tiangen Biotech Co., Ltd.). The resulting cDNAs were diluted 10-fold in nuclease-free water (Invitrogen). qPCR was carried out in a 20 μL solution including 10 μL 2*Power SYBR Green PCR Master Mix (Applied Biosystems), 2 μL diluted cDNA, and 300 nM gene-specific primers or 10 μL 2*miRcute miRNA premix (Tiangen Biotech Co.). These primer sequences are shown in Table 1. The relative mRNA or miRNA expression was calculated using the Pfaffl method [47] in which β-actin or 202 was used as an endogenous normalization control.

**Table 1:** Primers used for quantitative RT-PCR

Genes	Accession No.	Forward (5′-3′)	Reverse (5′-3′)	Annealing Temperature (°C)	Product size (basepairs)
**Nestin**	NM_016701	aactggcacctcaagatgt	tcaagggtattaggcaagggg	60	235
**Pax6**	NM_001244201	gcgcagacggcatgtatgata	gggttgccctggtactgaag	60	104
**Ki-67**	X82786	cagtactcggaatgcagcaa	cagtcttcaggggctctgtc	60	170
**Vimentin**	NM_011701	tggttgacacccactcaaaa	gcttttggggtgtcagttgt	60	269
**β3-tubulin**	NM_023279	cgagacctactgcatcgaca	cattgagctgaccagggaat	60	152
**Rhodopsin**	NM_145383	tcaccaccaccctctacaca	tgatccaggtgaagaccaca	60	216
**PKC-α**	NM_011101	cccattccagaaggagatga	ttcctgtcagcaagcatcac	60	212
**AP2α**	NM_001122948	gccgtccacctagccaggga	gattgggccgcgagttcccc	60	208
**Brn3a**	NM_011143.4	cgctctcgcacaacaacatga	ttcttctcgccgccgttga	60	121
**Calbindin**	NM_009788	ggcttcatttcgacgctgac	acgtgagccaactctacaattc	60	184
**GFAP**	NM_010277	agaaaaccgcatcaccattc	tcacatcaccacgtccttgt	60	184
**Rbm8a**	NM_001102407	gctctgttgaaggttggattct	gccccatcaaatcttgaccat	60	222
**β-actin**	NM_007393	agccatgtacgtagccatcc	ctctcagctgtggtggtgaa	60	152

### Cell viability

Cell counting (CCK8; Dojindo) was performed to evaluate RPC proliferation capacity. RPCs were seeded in 96-well plates at a cellular density of 1 * 10^4^ cells/well, and then the cells were transfected. After that, CCK8 solution was added to each well at 0 h, 24 h, 48 h, and 72 h of the culture period. These cells were incubated for another 4 h at 37°C. The absorbance was measured using an ELISA microplate reader (ELX800; BioTeK) at 450 nm. The viability was expressed as the A450 value [48] because cell viability is directly proportional to the absorbance at 450 nm.

### Statistical analyses

The data in this study are expressed as the mean ± the standard error (SE). Each experiment was performed at least three times unless otherwise specified. Statistical analyses were determined using the Student's *t*-tests or one-way ANOVA, and the difference was considered significant for a value of *P < 0.05.

## Abbreviations

RPCs: retinal progenitor cells
miRNA: microRNA
3′UTR: microRNA3′untranslated region
SE: microRNA3′untranslated regionstandard error
